# Management and Outcomes of Pulmonary Nodules in a Real-World Setting

**DOI:** 10.3390/diagnostics15131677

**Published:** 2025-07-01

**Authors:** Berta Mosleh, Pavla Sarova, Helmut Prosch, Joachim Widder, Clemens Aigner, Marco Idzko, Mir Alireza Hoda, Daniela Gompelmann

**Affiliations:** 1 Department of Thoracic Surgery, Comprehensive Cancer Center Vienna, Comprehensive Center for Chest Diseases, Medical University of Vienna, 1090 Vienna, Austria; berta.mosleh@meduniwien.ac.at (B.M.); clemens.aigner@meduniwien.ac.at (C.A.); mir.hoda@meduniwien.ac.at (M.A.H.); 2 Division of Pulmonology, Department of Internal Medicine II, Comprehensive Cancer Center Vienna, Comprehensive Center for Chest Diseases, Medical University of Vienna, 1090 Vienna, Austria; pavla.sarova@meduniwien.ac.at (P.S.); marco.idzko@meduniwien.ac.at (M.I.); 3 Department of Biomedical Imaging and Image-Guided Therapy, Comprehensive Cancer Center Vienna, Comprehensive Center for Chest Diseases, Medical University of Vienna, 1090 Vienna, Austria; helmut.prosch@meduniwien.ac.at; 4 Department of Radiation Oncology, Comprehensive Cancer Center Vienna, Comprehensive Center for Chest Diseases, Medical University Vienna, 1090 Vienna, Austria; joachim.widder@meduniwien.ac.at

**Keywords:** pulmonary nodule, pulmonary nodule management, high-risk pulmonary nodule, lung cancer, pulmonary metastases

## Abstract

**Background and Objective:** Due to the increasing use of imaging and lung cancer screening programs, the rate of detected pulmonary nodules has steadily increased over the past decade. Overall, the diagnosis and management of pulmonary nodules remain challenging. Moreover, no specific guidelines exist for the management of pulmonary nodules in patients with a history of previous malignancy. This study reflects the current management in a real-world setting in a specialized European center. **Methods:** In this retrospective single-center study, patients with a pulmonary nodule <3 cm referred to the Division of Pulmonology or the Department of Thoracic Surgery at the Medical University of Vienna, Austria, from November 2022 to July 2024, were analyzed. A subgroup analysis of patients with a history of previous malignancy was performed and compared to patients without previous malignancies. **Results:** In total, 356 patients (48.5% male, median age 67 years [IQR 61–74], 53.7% with a history of previous cancer) with a pulmonary nodule (mean size of 14.8 mm) were enrolled. Bronchoscopy, computed tomography (CT)-guided biopsy, or surgery was performed in 13.2%, 7.3%, and 65.2% of the cases, respectively. The overall malignancy rate was 70.5%. Pulmonary nodules in patients with a prior malignancy were significantly larger (*p* < 0.001), showed a progression in size (*p* < 0.001), and were found to be malignant more frequently when compared to patients without previous cancer (*p* = 0.032). **Conclusions:** As most patients referred to a specialized center represent a selected group of high-risk patients, the majority of pulmonary nodules were found to be malignant. In patients with a history of previous malignancy, tissue sampling is warranted as the rate of malignancy is high.

## 1. Introduction

Pulmonary nodules are well-defined or poorly defined rounded opacities on imaging measuring up to 3 cm in diameter [1]. Due to advances in computed tomography (CT) scan technology, the introduction of lung cancer screening programs, and the aging patient population, the number of pulmonary nodules detected has steadily increased over the past decade [2]. There are various guidelines, e.g., the Fleischner Society [3], British Thoracic Society [4], the American College of Chest Physicians [5], or the National Comprehensive Cancer Network [6] guidelines, that assist clinicians in the management of pulmonary nodules based on various factors, such as age, smoking status, nodule size, their radio-morphology as well as various validated nodule prediction models. However, the management pathway is not straightforward as the guidelines differ and do not cover all patient groups. These guidelines were developed before the widespread availability of modern imaging and biopsy techniques. Moreover, the choice of management strategy is influenced by patient comorbidities and preferences. Factors that guide the physician’s decision-making include nodule size, smoking history, age, history of previous malignancy, and blood tests [7]. Bigger nodule size is significantly more likely to receive an invasive sampling for histology, whereas older age limits recommendation for invasive testing. Based on clinical experience, clinicians’ assessment in terms of predicting malignancy in pulmonary nodules may be more accurate than previously validated nodule prediction calculators [8]. Therefore, the management of pulmonary nodules remains inconsistent and presents a significant challenge in everyday clinical practice wherein clinicians have to balance the benefit of invasive investigation for accurate diagnosis with the potential risks associated with tissue sampling.

A special subgroup that requires particular attention is patients with previously treated lung cancer or extrapulmonary malignancies, in which pulmonary nodules are more likely to be malignant. The pulmonary lesions may represent metastases but also primary lung cancer, as these patients have a higher risk of developing a second primary malignancy, the most common of which is lung cancer [9]. Therefore, the probability of the presence of metastases or primary lung cancer depends on the histology of the primary tumor. There is no consensus or specific guidelines regarding the management of pulmonary nodules in patients with extrapulmonary cancers, and clinical decision is made on a case-by-case basis [10,11,12]. The management of lung nodules in patients with prior malignancy is a pressing clinical need because of the high likelihood of metastasis or a new primary lung cancer. Investigating the current management of pulmonary nodules and their outcome in this specific high-risk patient population is an urgent clinical gap.

The aim of this retrospective study was to evaluate the management of pulmonary nodules in everyday clinical practice at a time when modern imaging techniques, advanced navigation bronchoscopy techniques, CT- and ultrasound-guided biopsy techniques, and minimally invasive surgical procedures are available.

## 2. Methods

This analysis is a single-center, retrospective, observational study that investigates the management of pulmonary nodules of patients referred to the Division of Pulmonology, Department of Internal Medicine II or the Department of Thoracic Surgery at the Medical University of Vienna, Austria, between 1 November 2022 and 31 July 2024. This study was approved by the Ethics Committee of the Medical University of Vienna (1867/2024; 11 October 2024) according to the Declaration of Helsinki. Written informed consent was waived due to the retrospective study design.

### 2.1. Patient Population

In total, 356 patients (48.5% male, median age 67, interquartile range [IQR] 61–74]) with a histologically unverified pulmonary nodule (mean size of 14.8 mm ± 6.6 mm) were included. Clinical data were obtained retrospectively from hospital databases, including radiology, bronchoscopy, surgical, and pathology reports. Images, including CT and positron emission tomography CT (PET/CT) scans, were reviewed, and radiology reports written by multiple radiologists as part of routine clinical management were collected. Data were recorded into an electronic data capture system from the first visit through follow-up and/or establishment of diagnosis. The nodule size and the radio-morphology (solid, cystic, part-solid ground-glass opacity [GGO], and pure GGO) on recent CT scans were reviewed. Patient characteristics (age, sex, smoking status, pulmonary function test (forced expiratory volume in 1 s [FEV_1_], diffusion capacity [diffusion capacity for carbon monoxide, single breath; DLCO SB], previous history of malignancy, size and location of the pulmonary nodule) were collected. The management of pulmonary nodules included follow-up CT scans, PET/CT scans, bronchoscopy (using the radial endobronchial ultrasound probe [EBUS] to perform endobronchial ultrasound-guided transbronchial biopsy [EBUS-guided TBB] under fluoroscopy, electromagnetic navigation bronchoscopy [ENB; Superdimension^®^] plus EBUS and fluoroscopy), transthoracic CT-guided biopsy, surgery (wedge resection, anatomical segmentectomy, lobectomy), or radiotherapy without proven tissue diagnosis. Diagnoses and treatment plans were discussed in the institutional interdisciplinary board of pulmonologists and radiologists and/or multidisciplinary tumor boards. In case of invasive testing, final histology was reported. When invasive and minimally invasive histological investigations were not feasible and a definitive diagnosis could not be obtained due to inaccessibility, the patient’s general condition and comorbidities, the pulmonary nodules without histological confirmation were classified as clinically benign or clinically malignant based on imaging features (morphologic characteristics, size, shape, density, presence of calcifications, enhancement, change in size, and growth rate) and clinical context: Pulmonary nodules with well-defined borders, calcification, and stability in size over two years were classified as clinically benign. Pulmonary nodules with spiculated and irregular borders, without calcification, with contrast enhancement or an SUV (standardized uptake value) uptake ≥ 2.5, and with enlargement in size on serial imaging, were classified as clinically malignant.

Pulmonary nodules were followed up for two years or until a definitive diagnosis was established.

Out of the 356 patients, 191 (53.7%) had a previous history of malignancy. The management of the pulmonary nodules in patients with previous lung cancer and with previous extrapulmonary malignancy was also investigated.

### 2.2. Statistical Analysis

Categorical data are presented as counts (n) and percentages (%), and scale variables are presented as median and interquartile ranges (IQR) or mean values ± standard deviations (SD) as appropriate. To evaluate differences in clinical data and risk factors between groups, absolute and relative frequencies were calculated for categorical variables and compared using a Chi-Square test. For continuous variables, the mean and standard deviation were calculated and compared using a *t*-test. For the skewed variables, data were dis-played as medians and interquartile ranges (IQR), and the post hoc Mann–Whitney U-test or the Kruskal–Wallis test was calculated to compare groups. Differences for all analyses were considered statistically significant for *p*-values < 0.05. Due to the exploratory character of the study, no adjustment for multiple testing was performed. Statistical analysis was performed and graphics were generated using the SPSS 30.0 software system (SPSS Inc., Chicago, IL, USA).

## 3. Results

### 3.1. All Patients with Pulmonary Nodules

In total, 356 patients (48.5% male, median age 67 years [IQR 61–74]) with pulmonary nodules (*n* = 402) were analyzed. The mean size of lung nodules was 14.8 mm (±6.6 mm). Based on the radio-morphologic characteristics, size, and growth rate in serial CT scans, and individual risk assessment with regard to history of smoking and previous malignancy, further clinical protocols included follow-up images or diagnostic biopsies. Patient demographics and lung nodule characteristics, as well as the detailed protocol of the clinical management, are described in Table 1.

Overall, 124 right upper lobe, 88 left upper lobe, 80 left lower lobe, 75 right lower lobe, and 35 middle lobe lesions were identified at the initial clinic visits. Most patients (n = 305, 85.7%) had solid nodules. Pure GGOs were seen in 20 patients (5.6%) and 17 patients (4.8%) had part-solid GGOs. The number of patients with cystic pulmonary lesions was 14 (3.9%).

Most of the patients (249/356, 69.9%) among the referred patients had a previous CT scan at the first presentation with a median interval of 5 months between the initial and follow-up scans. On these follow-up images, the pulmonary nodules were progressive in size, newly detected, or stable in 54.6% (136/249), 24.9% (62/249), and 19.3% (48/249) of patients, respectively. A minority of lesions (3/249, 1.2%) showed regression in size.

Therefore, invasive procedures as first diagnostic step after initial presentation were performed in a high proportion of all patients (71.9%, 256/356), including bronchoscopy, CT-guided transthoracic needle biopsy, or surgery in 40 (11.2%), 19 (5.4%), and 197 (55.3%) cases, respectively. All diagnostic surgical procedures were performed by a minimally invasive video-assisted thoracoscopic approach. Based on clinical and morphological risk assessment, surveillance, including CT or PET/CT scans, was conducted in 24.4% (87/356) of the cases. Stereotactic radiotherapy without verified histology was proposed in 13 (3.7%) patients at the time of first clinic visit.

**Figure 1:**
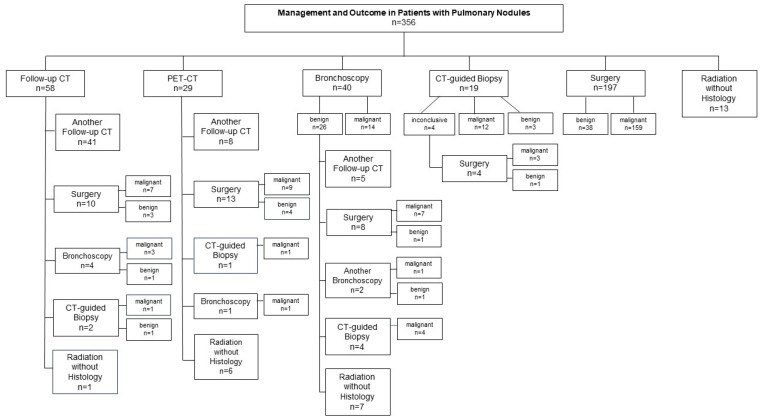
Management and outcome in all patients with pulmonary nodules. Surveillance CT or PET/CT scans were conducted in 24.4% (87/356) of the cases. As first diagnostic step, bronchoscopy, CT-guided transthoracic needle biopsy, or minimally invasive surgery was performed in 40 (11.2%), 19 (5.4%), and 197 (55.3%) cases, respectively. Stereotactic radiotherapy without verified histology was proposed in 13 (3.7%) patients. In the further course, histological diagnosis was established by bronchoscopy in 13.2% (47/356), CT-guided biopsy in 7.3% (26/356), or surgery in 65.2% (232/356) of the cases. Overall, a malignant diagnosis (histologically verified and clinically malignant) was found in 70.5% (251/356) of all patients.

In the further course, patients with inconclusive results underwent further procedures. Detailed management, as well as biopsy techniques and results, are presented in Figure 1. In total, to establish histological diagnosis, biopsy was performed by bronchoscopy in 13.2% of all cases (47/356; 72.3% EBUS-guided TBB, 27.7% ENB), while CT-guided biopsy was performed in 7.3% (26/356) of the cases. Ultimately, surgical resections (diagnostic and therapeutic) were performed in 65.2% (232/356) of the cases. 

Overall, a malignant diagnosis (histologically verified and clinically malignant) was found in 70.5% (251/356) of all patients. Malignancy was histologically confirmed (lung cancer or metastases) in 62.1% (221/356) of all patients. Bronchoscopy revealed malignancy in 40.4% (19/47) of the cases, while 59.7% (28/47) were found to be benign: the “benign” nodule was confirmed as benign in 32.1% (9/28; confirmed by surgery, another bronchoscopy, or follow-up CT scans) and inconclusive/false negative in 67.9% (19/28; malignancy confirmed by CT-guided biopsy or surgery or clinically suspected to be malignant). CT-guided biopsy diagnosed malignancy in 69.2% (18/26) of the cases (benign, 15.4% [4/26]; and inconclusive, 15.4% [4/26]), while surgery (wedge resection, 54% [126/232]; segmentectomy, 15.9% [37/232]; lobectomy, 29.7% [69/232]) established a malignant diagnosis in 79.7% (185/232) of all operated cases (benign, 20.3% [47/232]; and no inconclusive results). Histological confirmation of the pulmonary nodules was not provided in 22.8% (81/356) of all patients during the observation time, and in these cases, surveillance imaging or radiation therapy was proposed in 66.7% (54/81) and 33.3% (27/81) of the cases, respectively.

Significant differences with regard to malignant and benign diagnoses were found in age (*p* = 0.002), smoking history (*p* = 0.024), nodule size (*p* < 0.001), and change in size of the nodule (*p* < 0.001). In terms of clinical management, surgical biopsies (*p* < 0.001) and follow-up CTs (*p* < 0.001) were found to be significant measures to differentiate between benign and malignant disease (Table 2).

### 3.2. Patients with a History of Previous Malignancy

More than half of the patients (53.7%, 191/356) had a history of previous malignancy, of whom 33 (17.3%) had more than one previous neoplasm. The most common sites of previous cancers were carcinomas of the digestive tract, lung cancer, renal cell and urothelial carcinoma, gynecological malignancies, and breast cancer (Table 2). This subset of patients was significantly older than the group of patients without previous malignancies (*p* = 0.003). The pulmonary nodules were significantly larger (*p* < 0.001), were more likely to show a progression in size on serial CTs (*p* < 0.001), and were found to be malignant significantly more frequently when compared to the patient group without previous cancer (75% vs. 65%, *p* = 0.032). A detailed subgroup analysis between patients with and without a history of malignancy is presented in Table 3.

### 3.3. Patients with a History of Previous Lung Cancer

**Figure 2:**
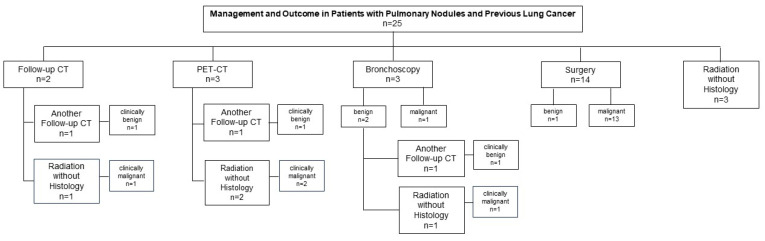
Management and outcome in patients with pulmonary nodules and a history of previous lung cancer. In patients with a history of previously treated lung cancer (25/356, 7%), follow-up CT or PET/CT scans were proposed in two (2/25, 8%) and three (3/25, 12%) cases, respectively. Biopsies were obtained by bronchoscopy in three patients (3/25, 12%; one malignant, one benign, and one inconclusive result). Surgery was performed in 14 patients (14/25, 56%; 13 malignant, 1 benign histology). Radiation without histological confirmation was proposed in three cases (3/25, 12%).

A total of 25 patients (25/356, 7%) had previously treated lung cancers. In this patient group with newly detected nodules (mean size 10.4 mm ± 4.3 mm) with a previous history of lung cancer, a follow-up CT scan was proposed in only two patients (2/25, 8%), while PET/CT scan was decided in three cases (3/25, 12%). Biopsies were obtained by bronchoscopy in three patients (3/25, 12%; one malignant, one benign, and one inconclusive result). Surgery was performed in 14 patients (14/25, 56%; 13 malignant results, including 12 lung cancers and 1 metastasis, 1 benign histology). Radiation based on individual risk assessment without histological confirmation was proposed in three cases (3/25, 12%). The details are presented in Figure 2.

### 3.4. Patients with Previous Extrapulmonary Malignancies

Out of 166 patients with a history of extrapulmonary cancer and a pulmonary nodule (mean size of 13.2 mm ± 5.6 mm), 58.4% of patients (97/166) underwent immediate surgical resection that confirmed malignancy in 82.5% of the cases (80/97; 57.5%, metastases; 42.5%, lung cancer).

Initial bronchoscopy was performed in 10.2% of the patients (17/166), revealing malignancy in 52.9% (9/17; metastases, 40%; lung cancer, 60%). In 47.1% (8/17) of patients with benign histology in the bronchoscopy biopsies, subsequent surgery (n = 2), another bronchoscopy (n = 1), and CT-guided biopsy (n = 1) confirmed lung cancer in 50% and metastases in 50% of the biopsied cases.

CT-guided biopsy was performed in 10 patients as first step. In 60% (6/10), histology revealed lung cancer, and in 40% (4/10), benign results were found. A subsequent surgery in these four patients, however, resulted in three lung cancer diagnoses and confirmed one benign lesion.

A follow-up CT or PET/CT scan was performed in 16.3% (27/166) and 5.4% (9/166) of the patients, respectively, followed by subsequent surgery in 30.6% (11/36), bronchoscopy in 8.3% (3/36), and CT-guided biopsy in 2.8% (1/36).

**Figure 3:**
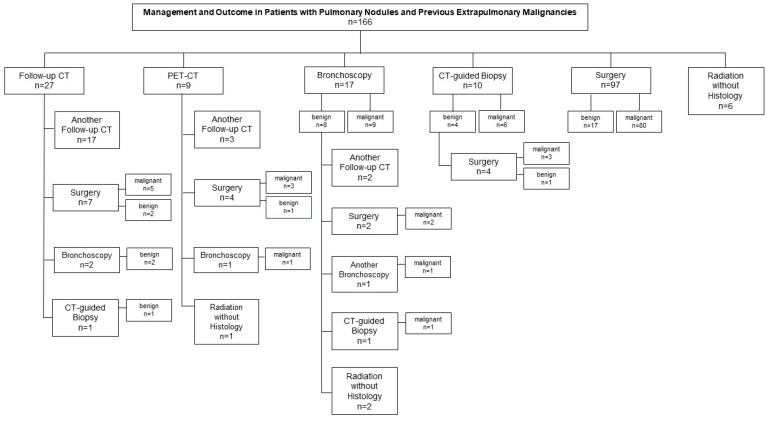
Management and outcome in patients with pulmonary nodules and previous extrapulmonary malignancies. Overall, a malignancy rate of 66.9% (111/166) was found. Out of 166 patients with a history of extrapulmonary cancer, 58.4% of patients (97/166) underwent immediate surgical resection that confirmed malignancy in 82.5% of the cases (80/97). Bronchoscopy was performed in 10.2% (17/166) of the patients revealing malignancy in 52.9% (9/17). In 47.1% (8/17) of patients with benign histology in the bronchoscopy biopsies, subsequent surgery (n = 2), another bronchoscopy (n = 1), and CT-guided biopsy (n = 1) confirmed malignancy. CT-guided biopsy was performed in 10 patients, in which malignancy was revealed in 60% (6/10), while in 40% (4/10), benign results were found. A subsequent surgery in these four patients resulted in three malignant diagnoses and confirmed one benign lesion. Follow-up CT or PET/CT scan was performed in 16.3% (27/166) and 5.4% (9/166) of the patients, respectively, followed by subsequent surgery in 30.6% (11/36), bronchoscopy in 8.3% (3/36), and CT-guided biopsy in 2.8% (1/36).

Overall, a malignancy rate of 66.9% (111/166) was found. Lung cancer or metastasis was detected in 33.1% (55/166) and 33.7% (56/166) of the patients with a history of previous extrapulmonary malignancies, respectively. Benign disease was found in 13.9% (23/166), while in 19.3% (32/166) of the cases, histological verification could not be obtained. The details are presented in Figure 3.

## 4. Discussion

This study describes the management of pulmonary nodules in patients in a real-world setting from 2022 to 2024. The patient cohort enrolled in this study is a prescreened patient population, as the patients were referred from community pulmonologists or other hospitals to a specialized university hospital for histological sampling of their nodules. Nearly 70% of patients had a serial CT scan on initial presentation, wherein the pulmonary nodules were progressive in size or newly detected in 80% of the cases. Changes in lung nodule size were found to be significantly associated with malignancy. This explains the high histologically proven malignancy rate of 62.1% in this study cohort, which is higher compared to other studies [13]. In particular, this patient cohort should not be compared to those of the lung cancer screening population: In the NELSON trial, a pulmonary nodule found in 1571 subjects out of 7582 patients in their first CT scan was found to be malignant in 5% [14]; in the LUSI trial, a pulmonary nodule was detected in 451 subjects out of 2028 in their first CT scan, among which 5% were diagnosed as lung cancer [15].

Given the very high probability of malignancy in this selected patient population, only 24.4% of patients were initially referred to another follow-up imaging, whereas in 71.9%, a histological sampling was immediately attempted. Indeed, a diagnostic and therapeutic surgical resection as first step was performed in 55.3% of patients, whereas non-surgical approaches (bronchoscopy or CT-guided biopsy) were only performed in 11.2% and 5.4%, respectively. In comparison to other studies, the surgical rate appears to be very high and the rate of non-surgical approaches low. Tanner et al. reported a rate of surgery as first step in 20.4% of patients and a bronchoscopy or CT-guided transthoracic biopsy in 33% [13]. This study, however, did not enroll patients with a prior diagnosis of any cancer within 2 years of nodule detection, with pulmonary nodules >2 cm in diameter, or the evidence of a progression in size of the nodule in serial CT scans. Therefore, the probability of malignancy in this study cohort was lower compared to our patient population, which may explain the less aggressive approach.

Yet, the rate of benign nodules detected by surgical approach was found to be 20% in our study, which was lower than in the study of Tanner and co-authors, who reported a rate of 35%. Nevertheless, a rate of 20% is still high considering that surgical interventions may be associated with higher complication rates compared to less invasive techniques. However, all surgical interventions were performed by a minimally invasive video-assisted thoracoscopic approach, and in 70% of all patients, the nodule was diagnosed by lung-sparing wedge resection or segmentectomy.

Bronchoscopy (EBUS-guided TBB under fluoroscopy and/or ENB + EBUS) or CT-guided biopsies for pulmonary nodules are associated with the risk of false-negative results. In our study, bronchoscopy in general revealed a malignant, benign, or inconclusive/false negative result in 40.4%, 19.2%, and 40.4%, respectively, resulting in a diagnostic accuracy of 59.6%. These rates are similar to those of other studies in which ENB was used for diagnosing peripheral lesions. In the prospective NAVIGATE study that evaluated the diagnostic accuracy of ENB for pulmonary nodules with a median lesion size of 20 mm, the malignancy rate was found to be 44.3% [16]. Out of the 55.7% benign results, 36.2% were considered false negative. Taking into account that the median size of the pulmonary nodules was even larger in the NAVIGATE study, the results are, therefore, comparable. Comparing our results with findings of a meta-analysis published in 2023 that reported diagnostic accuracies of EBUS-guided TBB, ENB, and EBUS + ENB of 70.9%, 74.0%, and 66.5%, respectively, our diagnostic accuracy was inferior [17]. This difference might also be attributed to the greater median lesion size in the included studies compared to our cohort. Besides electromagnetic navigation bronchoscopy, further navigation techniques are in fact available, among which the latest development is robotic-assisted bronchoscopy (RAB). To date, RAB is available in various centers in the United States, but only in a few centers in Europe. It could be hypothesized that the more widespread use of RAB will change the management of pulmonary nodules as a more effective first step in diagnosis, potentially reducing the rate of surgical biopsies. However, at present, this study reflects the bronchoscopic techniques available in most centers in Europe.

The diagnostic accuracy of CT-guided biopsy was superior to that of bronchoscopic tissue sampling (84.6% vs. 59.6%) according to findings of a meta-analysis that confirmed a superior diagnostic yield and diagnostic accuracy of CT-guided biopsies compared to EBUS-guided TBB [18]. However, the well-known limitations of CT-guided techniques, e.g., in foci that cannot be reached due to their location, extensive emphysema as a concomitant disease, and an increased risk of pneumothorax, are the reasons why bronchoscopic procedures are preferred in most cases.

Patients with a history of malignancy are a special group that requires particular attention. This group of patients was significantly older than the patients without previous malignancies. Moreover, the pulmonary nodules were significantly larger at initial presentation and were also more likely to show a progression in size on serial CTs. In addition, the pulmonary nodules were found to be malignant significantly more frequently when compared to the patient group without previous cancer (75% vs. 65%). The high malignancy rate in this subset of patients is supported by various studies. Khokhar et al. reported in a retrospective study that 42% of patients with extrapulmonary cancers were diagnosed with malignant nodules, of whom 50% had a newly diagnosed lung cancer, 44% had metastatic spread of their primary cancer, and 6% had a new neoplasm [11]. Qint et al. reported that 81.4% of pulmonary nodules detected in chest CT scans of patients with an extrapulmonary neoplasm were malignant [12]. Among these patients, most had a primary lung cancer rather than a metastasis or a benign lesion, although the likelihood of a primary lung cancer or metastasis varied depending on the presence of an extrapulmonary neoplasm. The knowledge of an increased rate of malignancy in this patient cohort may explain the immediate surgery in 58% of these patients. Therefore, in patients with prior malignancy, management decisions may be influenced both by clinical risk and by patient preferences for rapid histologic clarification.

The limitations of this study include the single-center design and the center’s high level of specialization with a highly selected patient population based on referrals for histological confirmation by external pulmonologists or specialists. Therefore, these results may not be generalizable to non-tertiary care settings.

In summary, a substantial fraction of patients with pulmonary nodules ≤3 cm in diameter, referred to a specialized center, ultimately proves to be malignant. In many cases, progression or persistence in nodule size on prior CT scans triggered immediate biopsy. In patients with prior malignancy, pulmonary nodules more frequently prove to be malignant, supporting early tissue sampling as the first step in management.

## Figures and Tables

**Table 1:** Patient and lung nodule characteristics.

Demographics	All Patients	Malignant	Benign	*p*-Value
Patients, n (%)	356	251 (70.5)	105 (29.5)	
Age, median (IQR)	67 (61–74)	68	64	0.002
Sex				0.110
Male	170 (47.8)	113	57	
Female	186 (52.2)	138	48	
Pack-years, mean	28	30	24	0.024
Smoking status				
Former	172 (48.3)	127	45	
Current	79 (22.2)	56	23	
Unknown	105 (29.5)	68	37	
Lung nodules				<0.001
Mean size, mm	15	16	12	
Nodule location *				
Left upper lobe	88	66	22	0.287
Left lower lobe	80	50	30	0.075
Right upper lobe	124	89	35	0.701
Middle lobe	35	24	11	0.070
Right lower lobe	75	51	24	0.592
Solid	305	210	95	0.450
Pure GGO	20	13	7	0.466
Part-solid GGO	17	2	15	0.153
Cystic	14	2	12	0.500
Dynamic of the nodule at first visit compared to previous images	249 (69.9)			<0.001
Regressive in size		0	3	
Constant in size		14	34	
Progressive in size		124	12	
Newly detected		45	17	
Diagnosis				
Benign	105 (29.5)	-	105	
Clinically benign (no histology)		-	51	
Inflammation/Infection		-	13	
Granuloma		-	22	
Hamartoma		-	13	
Intrapulmonary lymph node		-	2	
Amyloidosis		-	2	
Sarcoidosis			2	
Malignant	251 (70.5)	251	-	
Clinically malignant (no histology)		30	-	
Lung cancer		162	-	
NSCLC		159	-	
SCLC		3	-	
Pulmonary metastasis		59	-	
Management—first measures				
Biopsy				
Radial EBUS	28	22	6	0.330
Electromagnetic navigation	12	11	1	0.102
CT-guided biopsy	19	15	4	0.407
Surgery	197	159	38	<0.001
Radiation without histology	13	13	0	0.018
Follow-up CT scan	58	14	44	<0.001
Follow-up PET/CT scan	29	17	12	0.143
Management—next measures				
Biopsy				
Radial EBUS	6	4	2	0.835
Electromagnetic navigation	1	1	0	0.517
CT-guided biopsy	7	6	1	0.373
Surgery	35	26	9	0.606
Radiation without histology	14	14	0	0.014
Follow-up imaging	54	2	52	<0.001

* Nodule location refers to the total number of 402 nodules.

**Table 2:** Types of prior malignancies among patients with pulmonary nodules.

Site of Previous Malignancies	n (%) n = 224
Colorectal/appendix/small intestine/rectal/anal cancer	36 (18.8%)
Lung cancer	25 (13.1%)
Renal cell carcinoma/urothelial carcinoma	24 (12.6%)
Endometrial/ovarian/cervical/vaginal cancer	20 (11.0%)
Breast cancer	20 (10.5%)
Melanoma/squamous cell carcinoma/basal cell carcinoma of the skin	17 (8.9%)
Prostate cancer	15 (7.9%)
Pancreatic cancer/cholangiocellular carcinoma	13 (6.8%)
Head and neck cancer	12 (6.3%)
Lymphoma/leukemia/multiple myeloma	12 (6.3%)
Musculoskeletal tumors	11 (5.8%)
Esophageal/gastric cancer	7 (3.7%)
Thyroid cancer	4 (2.1%)
Testicular cancer	2 (1.0%)
Hepatocellular cancer	2 (1.0%)
Adrenal carcinoma	2 (1.0%)
Thymic cancer	1 (0.5%)
Chordoma	1(0.5%)

**Table 3:** Lung nodule management in patients without and with a previous history of malignancy.

Demographics	All Patients	No History of Previous Malignancy	With a History of Previous Malignancy	*p*-Value
Patients, n (%)	356	165 (46.3)	191 (53.7)	
Age, median (IQR)	67 (61–74)	65	68	0.003
Sex				0.420
Male	170 (47.8)	75	95	
Female	186 (52.2)	90	96	
Pack-years, mean	28	33	23	<0.248
Lung nodules				<0.001
Mean size, mm	15	17	13	
Dynamic of the nodule at first visit compared to previous images	249 (69.9)			<0.001
Regressive in size		3	0	
Constant in size		24	17	
Progressive in size		53	89	
Newly detected		6	57	
Diagnosis				0.032
Benign	105 (29.5)	57	48	
Clinically benign (no histology)		27	24	
Inflammation/infection		8	5	
Granuloma		12	10	
Hamartoma		9	4	
Intrapulmonary lymph node		-	2	
Amyloidosis		1	1	
Sarcoidosis		-	2	
Malignancy	251 (70.5)	108	143	
Clinically malignant (no histology)		12	18	
Lung cancer		95	67	
NSCLC		92	67	
SCLC		3	0	
Pulmonary metastasis		1	58	
Management—first measures				
Biopsy				
Radial EBUS	28	15	13	0.425
Electromagnetic navigation	12	5	7	0.741
CT-guided biopsy	19	9	10	0.927
Surgery	197	86	111	0.257
Radiation without histology	13	4	9	0.251
Follow-up CT scan	58	29	29	0.167
Follow-up PET/CT scan	29	17	12	0.542
Management—next measures				
Biopsy				
Radial EBUS	6	2	4	0.519
Electromagnetic navigation	1	1	0	0.281
CT-guided biopsy	7	5	2	0.179
Surgery	35	18	17	0.526
Radiation without histology	14	7	7	0.780
Follow-up imaging	54	29	25	0.239

## Data Availability

The data that support the findings of this study are available on request from the corresponding author.
